# Better Conversations With Parkinson's: Feasibility and Acceptability of a Novel Communication Partner Training Telehealth Intervention

**DOI:** 10.1111/1460-6984.70308

**Published:** 2026-08-04

**Authors:** Philippa Clay, Suzanne Beeke, Lucie Hogger, Ida Brix, Emma Massé, Hannah Hunt, Lynn Dangerfield, Steven Bloch

**Affiliations:** ^1^ Human Communication Science University College London London UK; ^2^ Wolfson Institute of Population Health Queen Mary University of London London UK; ^3^ Department of Nordic Studies and Linguistic University of Copenhagen Copenhagen Denmark; ^4^ Advanced Clinical Practice Professional Leadership Support Hampshire and Isle of Wight Healthcare NHS Foundation Trust Southampton Hampshire UK

**Keywords:** communication partner training, Parkinson's, telehealth

## Abstract

**Background:**

Communication Partner Training (CPT) has an established evidence base in aphasia, dementia and traumatic brain injury. It has been shown to improve the skills, attitudes and knowledge of the communication partner, and improve communicative participation and confidence of the person with communication difficulties. CPT therefore holds potential value for people living with Parkinson's and their everyday conversation partners who report significant impact of communication changes on their day‐to‐day lives. However, the CPT evidence base is less well established in this population. People living with Parkinson's and their communication partners report wanting support with everyday communication. They also describe online intervention as acceptable.

**Aims:**

The primary aim was to evaluate the feasibility and acceptability of a novel CPT intervention, Better Conversations with Parkinson's (BCP), when delivered via telehealth in an NHS setting. Feasibility was explored in terms of completion of BCP intervention and assessment data when delivered via telehealth, and the ability to recruit and retain participants in an NHS trial. Acceptability was investigated for: (1) dyads completing the therapy (people with a diagnosis of Parkinson's and their communication partners) and (2) the speech and language therapist (SLT) delivering it.

**Methods and Procedures:**

This feasibility study recruited dyads comprising a person living with Parkinson's and a familiar communication partner. Participants were involved for 6 weeks of direct BCP intervention, delivered remotely by one SLT, and completed pre‐ and post‐intervention measures. Descriptive statistics were used to report on recruitment, attendance, and attrition. Feasibility was further explored using logs and a focus group with study recruiters. Acceptability was evaluated using participant interviews, analysed using reflexive thematic analysis, and the SLT's reflective diary and interview feedback, which were analysed using qualitative content analysis.

**Results and Outcomes:**

Seven dyads were recruited to the study, with an overall recruitment rate of 10%. Participants completed all therapy sessions and data collection for all outcome measures was achieved. Qualitative themes reflected a high degree of acceptability from dyads concerning their own agency, the therapeutic alliance, methods used, and expectations/beliefs. The acceptability for the SLT delivering the therapy was also high.

**Conclusions and Implications:**

The results indicated the BCP programme was feasible in terms of online delivery within an NHS setting, and acceptable for clients and the SLT. These results pave the way for a large‐scale study that will evaluate the effectiveness of a BCP programme.

**WHAT THIS PAPER ADDS:**

*What is already known on the subject*

- Despite an identified need for treatments beyond the impairment level, communication partner training (CPT) for people living with Parkinson's lacks evidence for acceptability or feasibility.

*What this study adds to the existing knowledge*

- This study suggests that CPT for people living with Parkinson's is feasible to deliver via telehealth and acceptable to people living with Parkinson's, their communication partners, and SLTs.

*What are the clinical implications of this work?*

- UK NHS speech and language therapy services could feasibly run online CPT programmes for people living with Parkinson's. Further work is required to move toward a full‐scale trial of CPT for people living with Parkinson's and to establish whether such programmes may be most effectively run alongside impairment‐based approaches or independently.

## 1 Introduction

Parkinson's is the fastest‐growing neurological disorder (Ben‐Shlomo et al. 2024) and the second most common acquired neurodegenerative disease in the world (Ou et al. 2021). According to a 2018 Parkinson's UK report based on Clinical Practice Research Datalink primary care data from 2015, approximately 137,000 people in the UK were living with Parkinson's, a number projected to rise to over 168,000 by 2025 (Parkinson's UK 2018).

### 1.1 The Impact of Parkinson's on Speech and Communication

Hypokinetic dysarthric features are common in Parkinson's, typically characterised by a monotonous speech pattern, reduced loudness, a breathy or hoarse voice quality, an increase in speech rate, rapid repetition of phonemes, and imprecision in consonant production (Atalar et al. 2023). Additional communication challenges can result from interrelated changes in cognition (Rohl et al. 2022), language (Liu et al. 2015) and pragmatics (Montemurro et al. 2019). Self‐perception ratings suggest around 90% of people living with Parkinson's (plwP) experience some degree of speech‐language change (Miller 2017).

Conversations become difficult because of these communication changes, which often arise in combination with each other (Saldert and Bauer 2017). Communication difficulties related to Parkinson's impact on people's social communication (Sharma et al. 2023), and quality of life (Kavya et al. 2022). Furthermore, family members of plwP describe changes in family roles, decreased wellbeing and increased carer‐burden as a result of Parkinson's‐related communication difficulties (Mach et al. 2019). Communication difficulties in Parkinson's, therefore, are wide ranging with significant effects—both on plwP and their family members (Thilakaratne et al. 2026; Wylie et al. 2022).

### 1.2 Speech and Language Therapy Intervention

The evidence base for speech and language therapy for Parkinson's is predominantly focused around attention‐to‐effort based interventions such as Lee Silverman Voice Treatment (LSVT) (Ramig et al. 2001). LSVT has been shown to have significant impacts on volume and intelligibility in people with mild to moderate Parkinson's (Pu et al. 2021). However, speech approaches alone do not address the real‐world conversation‐based problems that plwP experience (Ferm et al. 2015), such as the timing of turn taking (Griffiths et al. 2012) or subtle issues arising from difficulties with word selection (Saldert et al. 2014).

In addition, speech approaches typically target the *individual* with Parkinson's, therefore failing to account for the unique needs of family members whose conversation is also affected (Baylor et al. 2024). The evidence is limited because those with more advanced Parkinson's symptoms are seldom included in communication intervention studies. Furthermore, whilst LSVT and other attention‐to‐effort therapies consider some cognitive aspects of communication alongside speech, the focus is on the impairment rather than participation, social interaction and the surrounding environment. There is a clear need, therefore, for evidence‐based speech and language therapy interventions that include family members, that are shown to be effective across the course of the disease, and that consider communication holistically.

### 1.3 Communication Partner Training

Communication Partner Training (CPT) is an umbrella term for complex behavioural interventions that focus on building the skills of a communication partner (family member, friend or partner), and sometimes the individual with a communication difficulty themselves, to enable successful and more meaningful conversations (Cruice et al. 2018). CPT approaches communication holistically rather than focusing on a speech, language or cognitive impairment in isolation, and is effective at improving the skills, attitudes and knowledge of the communication partner which in turn enables the participation of the individual with a communication difficulty (Simmons‐Mackie et al. 2016). The primary evidence for CPT is for people with aphasia (Best et al. 2016; Simmons‐Mackie et al. 2016), and CPT research has been growing for other conditions, particularly traumatic brain injury (Wiseman‐Hakes et al. 2020) and dementia (Nguyen et al. 2019; Volkmer et al. 2023). Despite the potential benefits of CPT, the evidence‐base for CPT with plwP remains limited (Thilakaratne et al. 2022). Case intervention reports (Bloch and Beeke 2021; Forsgren et al. 2013; Johansson and Müller 2026) show that CPT can be adapted and well received amongst people affected by Parkinson's (plwP and communication partners). However, these studies do not provide sufficient detail regarding acceptability and feasibility to drive intervention development and progress toward larger scale trials. Furthermore, Forsgren et al. (2013) evaluated CPT delivered only to the communication partner rather than dyad, and Bloch and Beeke (2021) and Johansson and Muller (2026) address face‐to‐face delivery only, despite the fact plwP find telehealth acceptable.

If shown to be effective, CPT could have a significant impact on the lives of many people affected by Parkinson's. Given the high incidence of Parkinson's, the prevalence of communication difficulties in this population, the range of communication difficulties experienced and the associated needs of family members, the need to move toward effectiveness studies in this area is pressing.

### 1.4 Telehealth

There is considerable evidence supporting the efficacy and acceptability of telehealth for plwP and SLTs (Maas et al. 2024; Theodoros 2021). There is strong evidence that plwP find telehealth satisfactory (Constantinescu et al. 2011), and evidence for the effectiveness of remote delivery of speech subsystem therapy exercises for Parkinson's (Quinn et al. 2019). One recent review of four trials investigating speech and language therapy delivery (including LSVT delivered to plwP) found no difference between telehealth service delivery and face‐to‐face delivery (Scott et al. 2025).

Research suggests that a telehealth approach to CPT is just as acceptable as face‐to‐face delivery to people with brain injury and their communication partners (Rietdjik et al. 2022), and that teleCPT improves access to therapy (Beeke et al. 2021). However, there is a lack of evidence for Parkinson's telehealth interventions that address day‐to‐day interaction and conversation.

### 1.5 The Better Conversations With Parkinson's (BCP) Study

This study evaluates the feasibility and acceptability of a novel teleCPT intervention for plwP: Better Conversations with Parkinson's (BCP). BCP is an adaptation of existing Better Conversations CPT, which has been found to reduce barriers in conversation and support people living with aphasia and dementia to achieve communication goals (Best et al. 2016; Volkmer et al. 2023). Better Conversations has also been delivered via telehealth as well as face to face (Beeke et al. 2021). In this study, BCP intervention was delivered to dyads entirely via telehealth, with the aim of improving access and extending the evidence for CPT with this population beyond face‐to‐face delivery.

An important phase in developing and evaluating complex interventions, alongside intervention development, evaluation and consideration of implementation, is the assessment of feasibility and acceptability of the intervention and evaluation design (Skivington et al. 2021). Feasibility studies explore whether something can be done, should we proceed to the next phase of research and if so, how (Eldridge et al. 2016). These studies therefore play an important role in identifying problems and preparing for a future randomised controlled trial (RCT). An exploration of feasibility is therefore the first step toward a future large‐scale trial to establish whether BCP is effective at improving the everyday day‐to‐day conversations of plwP and their communication partners.

In addressing feasibility, acceptability is a common focus (Bowen et al. 2009). As per the Theoretical Framework of Acceptability (TFA) (Sekhon et al. 2017), we viewed acceptability as a multi‐faceted construct, comprising component constructs: affective attitude, burden, perceived effectiveness, ethicality, intervention coherence, opportunity costs, and self‐efficacy. Acceptability can also be considered across different temporal perspectives: prospectively, concurrently and retrospectively, both from the patients’ and clinician's point of view.

To ensure that BCP is accepted by patients, possible to deliver within a trial and a clinical setting, and therefore readily implemented within the UK National Health Service (NHS), it was necessary to answer the following research questions:

1. How feasible is a full BCP study in terms of recruitment and retention within a UK NHS setting?
2. How feasible is completion of the BCP intervention and data collection for participants when delivered via telehealth?
3. How acceptable is BCP for people affected by Parkinson's?
4. How acceptable is BCP for SLTs?

## 2 Method

The research was overseen by a steering group of two plwP with communication difficulties, one family member and two SLTs (one specialist in neurodegenerative disorders and one professional lead).

### 2.1 Measures and Design

**FIGURE 1:**
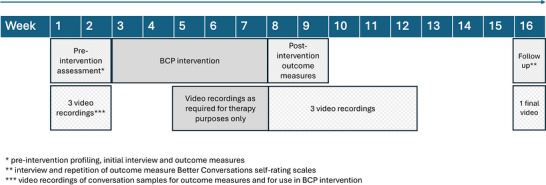
Study design overview.

Participants were involved in the study for approximately 16 weeks. See Figure 1 for an overview of the study design. The intervention was delivered by the first author, PC, a highly experienced SLT.

Pre‐intervention, PC interviewed participants to collect biographical data and understand a dyad's typical interactions, and to understand participant profiles using the Frenchay Dysarthria Assessment (Enderby and Palmer 2008), Montreal Cognitive Assessment (Nasreddine et al. 2005), and MDS‐Unified Parkinson's Disease Rating scale (Goetz et al. 2008). Pre‐ and post‐intervention measures (Table 1) examining functional communication, participation, feelings, knowledge about communication in Parkinson's, skills in managing day‐to‐day communication, and quality of life, were completed within the 2 weeks before starting the intervention and within 2 weeks of completing the intervention. The dyad made seven 15‐min video recordings with the support of PC: three times pre‐intervention, three times within 4 weeks of completing the intervention, and once approximately 2 months post‐intervention. Pre‐ and post‐intervention videos were used for analysis of conversation outcomes. Pre‐intervention videos were also used for video feedback within the therapy to support identification of facilitators and barriers to conversation (Beeke and Bloch 2023). Outcome measures were conducted pre‐ and post‐ the intervention, to examine the feasibility of assessment completion for a future large‐scale trial. Since refinement of the selected outcome measures is required prior to a future trial, outcomes are not reported as part of feasibility within this study.

**TABLE 1:** Pre‐ and post‐intervention measures.

Completed by:	Measure
Participant with a diagnosis of Parkinson's	Communicative Participation Item Bank (Baylor et al. 2013) Dysarthria Impact Profile (Walshe et al. 2009) The Voice Handicap Index (Jacobson et al. 1997) Parkinson's Disease Questionnaire (PDQ‐39) (Jenkinson et al. 1997)
Communication partner	Parkinson's Disease Questionnaire—Carers (PDQ‐Carer) (Jenkinson et al. 2012) Questionnaire investigating knowledge, skills and feelings around communicating with person with Parkinson's—adapted from the Health Professionals and Aphasia Questionnaire (Jensen et al. 2022) and Carer Communication Outcome After Stroke (Carer COAST) Scale (Long et al. 2009)
Both members of the dyad	Goal Attainment Scaling (GAS) in rehabilitation (Turner‐Stokes 2009)^a^ Better Conversations Self Rating Scales (Johnson 2011 unpublished) as described in Beeke and Bloch (2023)

^a^ Post‐intervention measure collected in Week 6 of intervention during the therapy session. All others collected post‐intervention.

### 2.2 Recruitment

Participants were recruited by their local SLT from active caseloads of a single community NHS site in England. At the time of the study, the collaborating NHS Trust estimated a speech and language therapy caseload of approximately 90 plwP experiencing communication and/ or swallowing difficulties. Recruitment was conducted in two phases (from July 2022 to January 2023 and from November 2023 to March 2024) due to personal circumstances of the SLT/ first author. During the second recruitment phase, participants were invited from the waiting list of the partner NHS Trust as well as from active speech and language therapy caseloads. It was also emphasised to participants that a speech and language therapy assistant could provide support to set up participants on video calls if required. For both recruitment phases, participants who did not have access to a computer were offered an iPad to borrow for Zoom sessions.

Recruitment ensured that plwP were supported to give fully informed written consent, as outlined in the published protocol (Clay et al. 2023). The aim was to recruit 10–12 dyads to the study, comprising an adult with a primary diagnosis of Parkinson's and related communication difficulties, and a communication partner (CP). Inclusion criteria (Supporting Information 1) stipulated that participants should be able to see and hear well enough to participate and be able to functionally engage in the programme. To avoid confounding factors, people were excluded if they had a history of brain lesions, had a major disability, behaviour or psychiatric diagnosis that would impact on participation, or if they were currently participating in communication therapy or research which might conflict with BCP. Participants were also required to have English as their language of daily use and access to Wi‐Fi.

### 2.3 Better Conversations With Parkinson's Telehealth Programme

BCP is a teleCPT approach co‐produced by four plwP, one family member, and four SLTs (one SLT, author PC, is a member of the research team) (see Clay et al. 2024). BCP involves the SLT working with a person living with Parkinson's and a regular conversation partner to build skills to improve day‐to‐day conversations. The therapy was delivered via an online platform (Zoom, version 7.0.2 (34412)) over six sessions. Initial sessions included setting expectations, education about holistic communication, reflection on what works well and less well in conversation and identifying personalised goals. The dyad then practised communication strategies in context and was supported to generalise skills and plan for the future. Home activities included self‐monitoring and reflection, planned use of strategies, and the option to record and send the SLT videoed conversations for additional feedback and reflection in the session. Further details of the programme are available under Supporting Information 2.

### 2.4 Evaluation of Feasibility and Acceptability

Feasibility was explored in terms of recruitment rates, time required to recruit to target, retention and attrition rates, session completion rates and video and outcome data completion rates. Reasons for declining to take part in the study, if voluntarily provided, were recorded by recruiting SLTs in an Excel spreadsheet to capture potential barriers to involvement. A focus group with recruiting SLTs was also used to explain and contextualise data on recruitment rates. The focus group (see Supporting Information 3 for topic guide) was run by the first author and another member of the research team well known to the recruiting SLTs (LD). All SLTs working with a neurological caseload at the recruiting NHS Trust were invited to take part.

To explore acceptability to those receiving the intervention, approximately 2 months post‐intervention dyads were interviewed by a student SLT (author H.H.). The semi‐structured interviews followed a topic guide (Supporting Information 3) and asked about original expectations and actual experiences of the therapy, barriers and enablers to completing therapy, and the impact of the intervention on their lives. Interviews also explored participants’ perceptions of the study and data collection to understand acceptability of the trial design more broadly. The topic guide was refined by the study steering group to ensure questions were accessible, relevant and appropriate. Participant interviews were conducted, recorded and transcribed by HH.

Given the importance of acceptability to the clinician to ensure fidelity of delivery (Sekhon et al. 2017), the perspective of PC, the SLT delivering the intervention, was also sought. After each session, PC completed written logs designed to examine concurrent acceptability. The logs asked about time required to prepare, the experience of delivering therapy, whether sessions met expectations or were adapted, what worked well or less well, and the perceived impact on the dyad. PC was also interviewed twice by two visiting student SLTs from Denmark (authors EM and IB) upon completion of therapy delivery, to explore prospective and retrospective acceptability. These semi‐structured interviews followed a topic guide (Supporting Information 3) based around the TFA framework (Sekhon et al. 2017) and investigated PC's expectations of the BCP programme, experiences of carrying out the intervention, and views on expectations of how BCP might fit within NHS delivery.

### 2.5 Analysis

**FIGURE 2:**
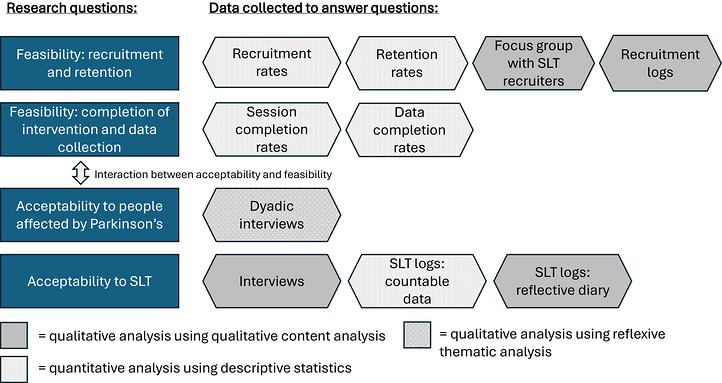
Overview of data collected and analysis used to answer research questions.

Recruitment and retention data were analysed using descriptive statistics. Reasons given around taking part in the study and focus group data were analysed using qualitative content analysis. Figure 2 outlines an overview of the data collected to answer study research questions, with corresponding analysis methods. The multiple data sources allow triangulation of data and different perspectives on multi‐faceted issues such as recruitment and acceptability.

The approach to analysing acceptability data differed for participant and SLT perceived acceptability. The diverse methods of analysis reflect the expertise across the team and the desire to explore participant acceptability with an inductive, reflexive approach. A deductive approach was judged appropriate for clinician data as the SLT (PC) was a member of the research team, and it facilitated full consideration of the construct of acceptability.

Reflexive logs were kept by HH, EM, IB, LH and LD to record potential subjective influences, preconceptions and analytical decisions. The analytical approach and themes were also discussed amongst the research team and with qualitative researchers to ensure themes were sufficiently distinct and multidimensional.

Participant interviews were analysed using reflexive thematic analysis (Braun and Clark 2006), using an inductive approach to identify deep meanings reflecting the experiences of participants. Given the use of the TFA for clinician acceptability, inductively identified themes related to participant acceptability were retrospectively mapped to the TFA.

The SLT interviews were analysed using deductive qualitative content analysis (Fife and Gossner 2024). For these interviews, predefined codes and categories were based on components of the TFA, with an additional category ‘other’ for information that fitted outside these components but was deemed relevant. Coding of SLT interviews was conducted by the authors E.M. and I.B. following pre‐established coding and rating guidelines. E.M. and I.B. coded independently, with overall inter‐rater agreement on 80% of codes in interview one and 97% of codes in interview two. E.M. and I.B. discussed discrepancies and resolved these through consensus.

Clinician logs were also analysed using deductive qualitative content analysis, using predefined codes from the TFA. The researcher (author L.H.) read each log several times. For the purposes of analysis, all logs across the seven dyads were collectively examined for each session at a time. Data were extracted to an Excel spreadsheet relating to yes/no answers, countable answers and scalable aspects of each session that the SLT rated (such as how easy it was to engage the dyad and make them feel comfortable). Clinician logs were used to triangulate the interview data and consider acceptability from different temporal perspectives as per the TFA.

## 3 Results

The results presented below aim to understand feasibility and acceptability of the intervention BCP.

### 3.1 Participants

Seven dyads were recruited to the study. Five of seven participants with a diagnosis of Parkinson's were male, in line with a higher prevalence of Parkinson's amongst men (Zirra et al. 2023). All communication partners were spouses or partners, in heterosexual relationships spanning 10–59 years. All participants (people with Parkinson's and communication partners) were White British. Using Index of Multiple Deprivation data (Ministry of Housing, Communities, and Local Government n.d), all participants came from neighbourhoods with low levels of deprivation.  All participants were educated to at least 16 years of age. Nine had or were pursuing further qualifications, five at degree level. Four participants remained in work, and 10 were retired.

In terms of disease and dysarthria profile, six of seven participants with Parkinson's had a diagnosis of idiopathic Parkinson's (Table 2). The remaining person (P7) had a diagnosis of atypical Parkinsonism. Time since diagnosis ranged from 1 to 12 years. The Hoehn and Yahr scale (Hoehn and Yahr 1967) and Movement Disorder Society‐ Unified Parkinson's Disease Rating Scale (Goetz et al. 2008) scores were used to judge severity of Parkinson's. Participants P2 and P3 achieved scores on the Montreal Cognitive Assessment (MoCA) (Nasreddine et al. 2005) which may indicate mild cognitive impairment. On initial assessment, four participants presented with mild dysarthria (P3, P4, P5 and P6), two with moderate dysarthria (P1 and P2) and one with severe hypokinetic dysarthria (P7). Five people with Parkinson's were on a waiting list for and had not previously received speech and language therapy.

**TABLE 2:** Parkinson's and dysarthria profile of participants.

	Parkinson's type	Years since diagnosis	MoCA	Hoehn and Yahr	MDS‐UPDRS I	MDS‐UPDRS II	MDS‐UPDRS III	MDS‐UPDRS IV	Dysarthria severity	Previous SLT input
P1	Idiopathic	12	28	2	18	33	67	8	Moderate	Yes
P2	Idiopathic	4	25	3	7	29	46	0	Moderate	No
P3	Idiopathic	1	12	2	4	6	30	2	Mild	No
P4	Idiopathic	4	28	2	17	25	29	2	Mild	No
P5	Idiopathic	11	29	2	22	21	29	11	Mild	No
P6	Idiopathic	2	26	2	15	15	27	7	Mild	No
P7	Atypical	3	26	4	15	36	71	4	Severe	Yes

*Note*: MDS‐UPDRS sections refer to: I—non‐motor experiences of daily living; II—motor experiences of daily living; III—motor examination; IV—motor complications.

The results relating to each research question will now be presented in turn.

### 3.2 Feasibility of Recruitment and Retention Within a UK NHS Setting

Feasibility was evaluated through recruitment data, with additional explanatory qualitative data from a focus group with recruiting SLTs and recruitment logs completed by SLTs. Recruitment occurred over a total of 10 months, during which seven participants were recruited. The overall recruitment rate for the 69 participants invited was 10%. Retention data were also collected. All seven recruited dyads were retained throughout the study.

Challenges in recruitment meant that the target of 10–12 dyads was not met. Recruitment rates for the two phases of recruitment are outlined in Table 3. In the first phase of recruitment, 34 people with Parkinson's and related communication difficulties were identified by recruiting SLTs, and 17 invited to take part in the study. Of those invited, one person with Parkinson's chose to take part with a communication partner, equating to a recruitment rate of approximately 6%. During the second phase of recruitment, 52 plwP were invited to take part in the study. The second recruitment phase included additional invitations to people on the waiting list and offers of support for setting up video calls. The majority (45) of these potential participants were on the waiting list for SLT input and invited via letter. Thirteen plwP replied to the invitation, of whom six then chose to take part in the study. The recruitment rate was, therefore, higher for this second phase of recruitment, at 11.5%.

**TABLE 3:** Recruitment rates for recruitment phase one (6 months), phase two (4 months, with additional recruitment from waiting lists) and overall.

Recruitment phase	Number invited	Number participated	Recruitment rate
1	17	1	6%
2	52	6	11.5%
Total	69	7	10%

SLTs documented some reasons for people not being invited to the study related to inclusion criteria, such as “no communication difficulties”, “change of diagnosis” and “no communication partner”. There were also reasons documented that did not align with the exclusion criteria, such as “can't access video calls”, “anxiety”, “psychological trauma”, “moved to care home” and “carer stress”. SLTs also briefly documented the perspectives of 23 potential participants who declined to take part. Example quotes from the SLT log are given in brackets. The three most frequently stated reasons for declining related to not having a communication partner available (“wife is too busy”), lack of confidence with telehealth delivery (“not good with technology”) and concerns about the level of input or time required (“too much commitment”). Other reasons raised related to health difficulties that precluded either the plwP (“cognitive difficulties too significant”) or the communication partner (“has visual difficulties”) taking part. One individual also chose not to take part due to the delay in receiving local speech and language therapy input.

A focus group with recruiting SLTs was held to understand and contextualise recruitment rates. Two SLTs attended who were significantly involved in recruitment. Focus group discussion about reasons for not including participants and reasons for people declining to take part echo the reasons described above. SLTs mentioned people having cognitive difficulties, not having a communication partner, or people not perceiving themselves as having communication problems that require intervention. SLTs felt that participant responses to taking part in the research varied: some were enthusiastic about contributing to research and accessing therapy sooner than usual; others were deterred by technology requirements, time commitments, or anxiety about being recorded. SLTs noted the difficulty in finding potential participants in the “sweet spot”—neither too severely affected, nor too mild.

Looking ahead, therapists identified several learning points. These included the need for clearer resource allocation agreements from the outset and potentially being less restrictive about cognitive criteria. They valued the support from the research team and saw the intervention as a potentially valuable addition to their service offering, though recruitment numbers were lower than they had anticipated when first reviewing the caseload for numbers of people with a diagnosis of Parkinson's.

### 3.3 Feasibility of Completion of BCP Intervention and Data Collection via Telehealth

Completion rates of the BCP intervention and assessment data were high. Dyads attended all six intervention sessions. All seven plwP and their communication partners completed pre‐intervention measures and post‐intervention measures in their entirety. Despite reported difficulties making videos (which participants could do independently or via teleconference with the SLT), all seven dyads recorded the required number of 15‐min videos. All dyads completed the post‐intervention interview. To facilitate completion of outcomes, reminders were sent via email, and participants could complete online forms at their convenience.

### 3.4 Acceptability to People Affected by Parkinson's

**FIGURE 3:**
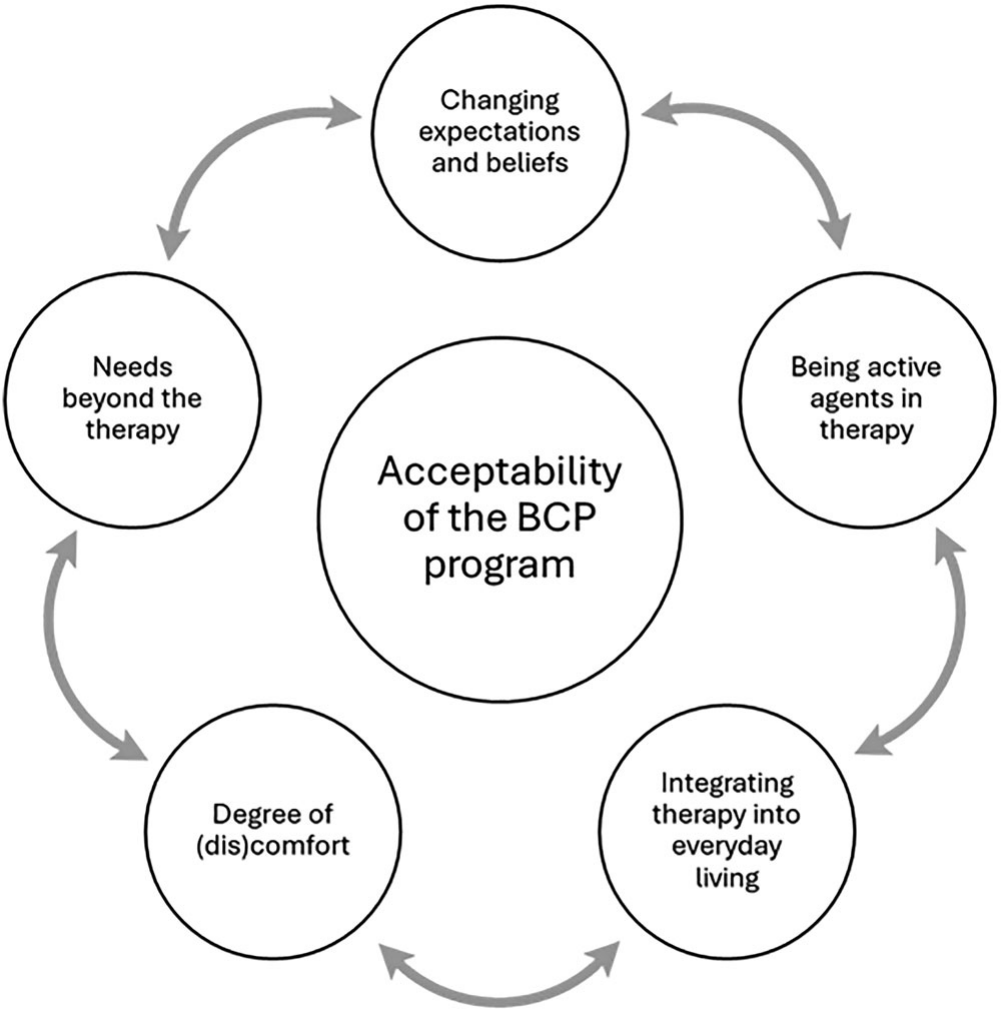
Acceptability of the BCP programme: five themes.

Acceptability to participants was evaluated using dyadic interviews conducted approximately 2 months post therapy. BCP was judged to be acceptable to participants. Five themes (Figure 3) represented distinct dimensions of participants’ experiences of the BCP programme that contributed to its perceived acceptability. These were the process and importance of changes to participants’ expectations and beliefs around what therapy offers (theme 1), participants being active agents in therapy (theme 2), the ease with which therapy was integrated into participants’ daily lives (theme 3), the degree of (dis)comfort participants experienced during therapy (theme 4) and the extent to which participants identified needs beyond therapy (theme 5). The views of people with Parkinson's (P) and communication partners (CP) are summarised below.

#### 3.4.1 Theme 1: Changing Expectations and Beliefs

Having entered the programme with expectations shaped by previous experiences of or knowledge about speech and language therapy, most participants anticipated voice exercises. However, all participants felt the SLT made the scope of therapy clear in the first session.

Most participants came to value the programme's emphasis on broader communication strategies and interpersonal dynamics. One dyad felt the programme was more useful than “physical” work would have been: P4 reflected, “I just thought it would be about volume or something similar, and it wasn't, it was far more than that.”

All participants expressed that conversations got “a bit” or “much” easier following therapy. Other positive changes included participants feeling “optimistic that we could continue to enjoy socialising” (CP5) and reduced tension within their couples.

For some, as their understanding of the therapy's aims and their communication habits evolved, so too did their recognition of the therapy's relevance to them, suggesting therapy contributed positively to participants’ insight and engagement.

#### 3.4.2 Theme 2: Being Active Agents in Therapy

Participants consistently described themselves as active contributors within a collaborative therapeutic process. They viewed the SLT as a facilitator who supported them to identify problems, set goals and generate solutions. CP7 explained, “we were doing the problem‐solving really, she was just asking the right questions and guiding us”. This highlights how a collaborative therapeutic alliance enhanced participants’ ownership of change.

Additionally, taking an active role in therapy appeared to increase self‐efficacy. For example, CP3 claimed, “it's just given me the confidence that I know what to do.” Furthermore, it enabled participants to tailor sessions around issues that held personal significance. As the partner of someone with communication needs, CP1 particularly appreciated being actively included in therapy, as this meant having her concerns and needs met as well as her partner's:

Normally if [P1]’s had speech therapy, I've just sat to the side and perhaps listened … but I was actually involved in this … which was good because it was solving a problem for both of us in a way.

However, the sense of responsibility that came with being active participants in therapy also brought challenges. Some participants reported lapses in motivation or inconsistent application of strategies and attributed setbacks to their own lack of effort.

#### 3.4.3 Theme 3: Integrating Therapy Into Everyday Living

Participants framed therapy as one amongst many tasks to complete within their already busy lives. Competing priorities included working, managing a household, travelling and socialising. Furthermore, participants proposed that living with Parkinson's introduced additional demands on their time and energy, such as exercising and attending appointments. In light of this, participants expressed a reluctance to sacrifice much more to the condition: “we try just to live our lives and the Parkinson's is kind of there, but it's not, we try to keep it being not a big deal” (CP4). Participants therefore evaluated the acceptability of the BCP programme in terms of how well it fitted into their pre‐existing routines and the costs of participation.

In general, teletherapy was reported to be convenient, accessible and efficient. Cited benefits of teletherapy were not having to travel to appointments or host the SLT, which were described as effortful and time‐consuming: “it [telehealth] is a time saver … it was preferable I'd say” (CP7). Crucially, Zoom was also described as “simple” to use (CP4), with session structure enhancing ability to participate (P1). The few obstacles that were reported, including computer audio feedback (P2) and difficulties uploading videos (CP4) were promptly resolved. The SLT's technological competence and virtual facilitation skills in making sessions accessible was felt to be important.

Despite live online sessions being generally regarded as convenient, participants still experienced challenges which somewhat compromised compliance with the programme. For example, several participants described struggling to prioritise reading and practising strategies outside of sessions amidst other commitments. There were also mixed views on therapy dosage. Whereas dyad 6 found the number of sessions excessive, suggesting they were able to put strategies “immediately into play”, P1 and P7 described discovering “something new” and of “value” in each session.

Finally, although teletherapy was deemed practical, a tension between valuing the quality of experience versus convenience was noted in participants' accounts. P3 remarked that being online was “not something I'm particularly comfortable with but it made it simpler.” Similarly, dyads 4 and 5 acknowledged there was something “nice” about meeting their SLT in person for assessment before the programme started which online meetings did not deliver.

#### 3.4.4 Theme 4: Degree of (Dis)comfort

The acceptability of BCP to participants was influenced by their enjoyment of sessions. Many participants claimed their rapport with the SLT facilitated openness and engagement with the program: “we felt we had a good relationship with her, so we opened up to her about things perhaps we might not have done to somebody that we didn't feel comfortable with” (CP3).

It seems participants’ enjoyment of sessions was also partly dependent on their personalities and dynamic as a couple. CP6 appreciated sessions as they necessitated opportunities for her and her partner to interact meaningfully: “we had to engage in a proper conversation because it was necessary for being recorded”. Nevertheless, engaging in the BCP program was not always easy for participants. The use of video was the most reported cause of discomfort. For example, P1 said, “we found the recordings very painful … but very useful”. Another complaint was that sessions were tiring, especially for those experiencing cognitive fatigue related to Parkinson's: “for me to concentrate on it for an hour is quite tricky, it's tiring” (P3). Others reported feelings of discomfort when engaging with the topic of Parkinson's. One participant (P2) also seemed to experience frustration or disappointment at not being able to contribute as they wished during sessions: “I didn't feel I was contributing as much as she [the SLT] was expecting … I really couldn't join in as I wanted”.

However, participants often recognised the value of these challenging aspects, suggesting that discomfort did not necessarily undermine acceptability—rather, discomfort was part of a meaningful and reflective process. This was especially prevalent in discussions about the use of video and fatigue: “I think it's exhausting because we were involved in it and that's when you put more adrenaline into it, when you get involved … it's not a criticism at all” (P1).

#### 3.4.5 Theme 5: Needs Beyond the Therapy

Even though BCP was generally thought to be beneficial, not all participants found the BCP program sufficient for their needs. Several participants confessed that they were still seeking “traditional speech therapy”. CP5 also suggested that the offer of BCP alongside voice exercises would appeal.

Moreover, one dyad expressed persistent disbelief in BCP's relevance or potential effectiveness for them. P2 stated “in my case it's not helped very much”, and CP2 reflected “there's no way his speech was going to improve during it… nothing can improve that to be honest”. This signals that the dyad may have been measuring progress using the metric of speech intelligibility rather than communication. These reflections reinforce the significance of participants’ personal contexts and alignment of expectations and beliefs regarding therapy outcomes in shaping acceptability, as illustrated in theme 1.

For some participants, BCP could not provide the type of support they felt they most needed. For example, CP6 alluded to feeling isolated and unsupported, suggesting that the programme did not address her emotional, social or care needs. Although it may be beyond the scope of BCP to provide significant emotional or social care support, this highlights the importance of individual fit and the need for holistic, integrated care systems. Although BCP was acceptable and beneficial for most participants, it was not universally sufficient, underscoring the value of offering it alongside other services.

These inductively identified five themes mapped closely onto the seven constructs of the TFA. This is evidenced in Supporting Information 4.

### 3.5 Acceptability to SLTs

SLT acceptability was evaluated through two retrospective interviews with the SLT delivering the intervention and concurrent logs completed by the SLT after each of the six sessions across the seven dyads. Prospective acceptability was also evaluated where possible from the SLT's interview responses. Results from the interviews and clinician logs are presented below, in alignment with the TFA, with each construct indicated in italics. Interview quotes exemplifying the prospective and retrospective perceptions of the SLT related to the TFA components can be seen in Supporting Information 5.

Under the construct of *affective attitude*, the SLT expressed positive emotions about the programme's promise both before and after therapy. The SLT valued the fact that the intervention can be tailored to individuals and saw potential to use it flexibly alongside impairment‐based interventions. Before delivering the therapy, the SLT felt that the likely *burden* would relate to preparation for the video feedback element of therapy, in terms of locating relevant video clips, preparing clips for use in therapy sessions and storing the data safely. This aligned with her perceptions retrospectively. In addition to this, SLT logs revealed burden of effort required within the session. This was associated with time management to cover all planned activities and content prioritisation when time was limited. The SLT evaluated the burden related to delivery of therapy and use of video as acceptable, emphasising that the process of video analysis and storage became quicker over time.

In terms of *ethicality*, after therapy delivery the SLT felt the therapy fitted with personal values: considering communication holistically, supporting people to make changes that affect their everyday lives, and including family members in the therapy process. Considering next *intervention coherence*, the SLT indicated high levels of understanding of the intervention and insight into the programme principles, both before and after delivering the therapy. Knowledge was also gained whilst using the intervention, which enabled more flexible use of the programme. The SLT's views relating to *opportunity costs* were specific to the delivery of the intervention in a research setting, as the SLT felt some plwP may have benefited from a different intervention approach at that time. When asked to consider *perceived effectiveness*, the SLT indicated that she had expected participants to make progress toward their individual goals, and that she felt this was achieved. However, not all improvements were as measurable as anticipated, for example, when dyads targeted conversation strategies that were not readily observable in a conversation video recorded for outcome measurement purposes. The SLT described gaining confidence in delivering the therapy over time, with increased ease at delivering the therapy more flexibly as familiarity with the intervention grew, which relates to the construct of *self‐efficacy*. Overall, on the basis of the SLT's sustained confidence and growth, delivery related to self‐efficacy was reported as acceptable, despite challenges related to management of emotional, relational and family needs within sessions in the context of online delivery.

In summary, BCP was acceptable to the SLT across all TFA components, from prospective, concurrent and retrospective viewpoints. Cohesion between these temporal perspectives suggests that the experience of delivering the intervention met expectations. The SLT's logs showed high acceptability in particular across *affective attitude, intervention coherence* and *perceived effectiveness*.

## 4 Discussion

The evidence for feasibility and acceptability of the teleCPT programme BCP is discussed below with reference to the study aims. Our first aim was to establish BCP feasibility in an NHS setting. The 10% recruitment rate for dyads from one NHS Trust appears low although this does compare favourably with other CPT studies (Volkmer et al. 2023). Despite this, retention and completion rates were high. There is little doubt that a full‐scale study across multiple NHS sites would be feasible given the numbers of plwP, and robust evidence from other recent large‐scale speech and language therapy Parkinson's intervention studies in the UK (Sackley et al. 2024), but there are three areas that may demand further attention. Firstly, the inclusion criteria relating to cognition may have prevented some SLTs from identifying eligible participants. Secondly, there was a mismatch between reasons for not inviting participants (such as anxiety, carer stress) and the inclusion criteria. Thirdly, there is a lack of dyad confidence or experience with telehealth. The first two areas may be addressed through more dialogue with referring SLTs and a more appropriate way of classifying cognition as part of the participant identification process. The latter may be mitigated by adopting a blended intervention in which there is at least some opportunity for face‐to‐face engagement at the start of the BCP programme.

Our second aim was to establish the degree of acceptability amongst plwP and their communication partners. Telehealth was considered convenient and a practicable mode of delivery. This supports existing evidence relating to speech and language therapy and Parkinson's (Maas et al. 2024; Theodoros 2021). However, the fact that the benefits of face‐to‐face interaction was also raised suggests a need to consider a blended approach that accommodates both convenience and human closeness (de Gier et al. 2023). Such an intervention package could, for example, be delivered predominately online, but beginning and ending with face‐to‐face engagement.

Expectations of the therapy were broadly in line with those set out by the SLT at the start of the BCP programme, but the desire for direct voice/volume therapy was also apparent. It is perhaps inevitable that plwP, their communication partners, and indeed SLTs will come with different beliefs and expectations about what speech and language therapy can offer. The existing evidence base is firmly grounded in studies of the efficacy of impairment based approaches such as LSVT (Xu et al. 2020), whereas the evidence base for interpersonal communication interventions in Parkinson's is at a much earlier stage of development (Bloch and Beeke 2021; Forsgren et al. 2013; Johansson and Müller 2026) although strong for other conditions such as aphasia (Simmons‐Mackie et al. 2016). Participants’ realisation that BCP focused on conversation and could be impactful was pivotal in encouraging acceptance and engagement. This echoes research which emphasises the centrality of intervention coherence and perceived effectiveness to acceptability and success (Baldwin et al. 2007; Sekhon et al. 2017).

Throughout participants’ accounts, there was also a sense that BCP's emphasis on collaboration between participants and the SLT, which is designed to promote self‐efficacy and self‐management, enhanced acceptability. This aligns with previous research, which indicates that plwP and their families value being involved in healthcare decisions and therapy being tailored to their specific needs (van der Eijk et al. 2011; Yorkston et al. 2017b).

The overall impression from the data was that most participants perceived BCP to be beneficial, but not universally appropriate or sufficient. Yorkston et al. 2017a p.216) noted that plwP who had undergone direct voice therapy felt that while it had equipped them with a useful tool, their toolbox still felt incomplete. This sentiment closely mirrors the experiences of participants in the present study, many of whom indicated that BCP only partially addressed their communication and wider needs. These reservations likely reflect a broader lack of emotional and social support for plwP and their caregivers—a well‐documented issue in the Parkinson's literature (Aamodt et al. 2024; Perepezko et al. 2019; Yorkston et al. 2017a).

One further identified issue was how participants can integrate BCP into their lives. This includes the number and length of sessions, and how much work outside of sessions is feasible. One factor that diminished the perceived acceptability of BCP was the interaction with complex emotional needs or entrenched self‐limiting beliefs. Those who exhibited caregiver burden or coping styles which were not conducive to engaging with BCP appeared to report lower satisfaction with therapy duration and outcomes. This is in line with research into CPT for aphasia, which suggests the importance of considering partner coping style and outlook on caregiving when selecting candidates (Wielaert et al. 2016).

Our final aim was to establish the degree of SLT acceptability. Consideration of SLT acceptability expands upon previous CPT studies for plwP (Bloch and Beeke 2021; Forsgren et al. 2013; Johansson and Müller 2026), which have predominantly focused on acceptability for those receiving the intervention. The SLT in this study found delivery of the BCP programme acceptable in all key areas. The results therefore align with SLTs’ positive perceptions when delivering Better Conversations with Primary Progressive Aphasia (Volkmer et al. 2023). The need for increased sessions raised in Volkmer et al.’s study was not an issue to the SLT delivering BCP, perhaps because of the increased number of sessions in BCP (six rather than four) or differences between populations.

The main challenges to SLT acceptability in this study pertained to burden. This was considered in two ways: preparation prior to the session, and the burden of effort required within the session. Within session burden was associated with time management to cover all planned activities, and content prioritisation when the clinician had to make decisions about what to include given limited time. This may be mitigated by refined session planning guidance and clearer prioritisation of key content within each session. A second challenge related to self‐efficacy, centred around management of emotional, relational and family needs within sessions in the context of online delivery. This may be addressed by supporting clinicians to continually develop skills and confidence in counselling techniques.

### 4.1 Study Limitations

A significant limitation to this study is the fact that only one SLT was involved in the delivery of the intervention. Furthermore, as the research assistant on this study, she was heavily involved in the development of BCP. These two factors introduce an obvious risk of bias that needs to be addressed in future work. It is also notable that there were recruitment challenges and only seven dyads (14 participants) from one NHS Trust participated in the study. Furthermore, the dyads’ demographic characteristics and communication profiles of the plwP do not reflect the heterogeneity of the population living with Parkinson's. Whilst a small sample size at a single site is appropriate to considering specific questions of feasibility and acceptability, this limits transferability of results. In preparation for a full‐scale trial, future work will need to target multiple sites and a more heterogeneous population, and implement learnings in relation to recruitment, for example, through refinement of inclusion criteria.

### 4.2 Next Steps in Research

Having evidenced initial acceptability and feasibility of the BCP programme, an additional next step will be to finalise robust outcome measures that capture the intended behaviour change targeted by the intervention. Work is ongoing to address this. There may also be value in exploring how both CPT and voice/volume interventions are able to complement each other. Moving toward an appropriately scaled RCT in which BCP is compared with UK NHS therapy as usual is also an important evidence objective.

## 5 Conclusions

This study is the first to demonstrate feasibility and acceptability of a telehealth CPT programme for plwP. The results suggest high levels of acceptability and feasibility of BCP for participants and for the delivering SLT. We have identified important areas for future development which will support the continued design and evaluation of this complex behavioural intervention for plwP and for their communication partners.

## Ethics Statement

Ethical approval for the study was granted by the London—Central NHS Research Ethics Committee (Ref: 22/LO/0332) in May 2022.

## Consent

Written informed consent, including for the publication of non‐identifiable data, was obtained from all participants in this study.

## Conflicts of Interest

There are no potential sources of conflict of interest.

## Supporting information




**Supporting Information**: jlcd70308‐supp‐0001‐SuppMat.docx

**Supporting Information**: jlcd70308‐supp‐0002‐SuppMat.docx

**Supporting Information**: jlcd70308‐supp‐0003‐SuppMat.docx

**Supporting Information**: jlcd70308‐supp‐0004‐SuppMat.docx

**Supporting Information**: jlcd70308‐supp‐0005‐SuppMat.docx

## Data Availability

The data that support the findings of this study are available from the corresponding author upon reasonable request.
