# Supplementary material for: Better Conversations With Parkinson's: Feasibility and Acceptability of a Novel Communication Partner Training Telehealth Intervention
Source: Int J Lang Commun Disord. 2026 Aug 4;61(5):e70308. doi: 10.1111/1460-6984.70308 (PMC13437927; doi:10.1111/1460-6984.70308)
Supplement: Supplementary file 1 [file JLCD-61-0-s001.docx]

## Supporting Information 2: Template for Intervention Description and Replication (TIDieR) Checklist for Better Conversations with Parkinson’s

The Better Conversations with Parkinson’s (BCP) programme is described below, using the template for intervention description and replication (TIDieR) checklist (Hoffmann et al., 2014).

| Item Number | Item |
| --- | --- |
| 1. | **Brief name**  Better Conversations with Parkinson’s (BCP) |
| 2. | **Why**  Better Conversations with Parkinson’s (BCP) is an approach to communication partner training that aims to help people with Parkinson’s have more successful interactions and conversations in their everyday lives. It involves working with people with Parkinson’s and their regular communication partners as a pair or ‘dyad’. The therapy involves developing the best strategies for their conversations as a dyad, through:   - increasing knowledge of conversation - understanding how their dysarthria affects conversation and participation - identifying barriers and facilitators to conversation - practising chosen strategies in conversation, coached by the Speech and Language Therapist (SLT)   The approach aims to identify, reinforce and build on the dyad’s current communication skills, and to allow them to identify their own barriers and preferred strategies to encourage ownership and self-management. By working with people with Parkinson’s and their partners, the approach also aims to enable more sustainable change in real life situations.  The approach is based on the principles of a Better Conversations approach to communication partner training. The Better Conversations approach incorporates theories of behaviour change (Johnson et al., 2017), for example providing multiple opportunities for people to reflect on and practise strategies, using video feedback to support reflection, and focusing on elimination of barriers as well as implementation of facilitative strategies. Self-efficacy and self-management are promoted through principles such as focusing on patient-centred problems and goals (Yorkston et al., 2017), and using problem solving to address barriers to conversation. |
| 3. | **What**  Materials:  Prior to the intervention, dyads are asked to record three 15-minute conversations, which are used to provide short clips of video feedback during therapy sessions.  Each therapy session is accompanied by a session plan as a guide for the SLT, and handouts and homework for the dyad completing the therapy. There is a website (currently only available to study participants) which hosts information, videos and handouts for those taking part in the therapy. These can be accessed within and between sessions. At the end of each session, the SLT provides a written summary for the dyad. Following is a summary of the materials currently used (website resources, session plans, activity sheets for use in the session, information sheets/ handouts and home activity sheets):  Background information:   - Webpage: About Better Conversations, including video ‘Introduction to Better Conversations’ - Webpage: Communicating with Parkinson’s, including videos ‘How does Parkinson’s impacts on communication?’ and ‘Why work on conversation?’ - Webpage: Speech and Language Therapy for people living with Parkinson’s, including video ‘A SLT’s perspective of Better Conversations’ - Webpage: Your therapy sessions, including videos ‘Why work with a conversation partner?’ and ‘Your therapy programme – what to expect’   Session 1:   - Session plan 1: Understanding how conversation works - Webpage: Session 1, including information sheets below, key terms and video ‘what is conversation?’ - Information sheet: Understanding how ‘speech’ and ‘conversation’ are connected - Information sheet: How does conversation work - Information sheet: What makes conversations go well - Home activity: Identifying what works well and what challenges exist in conversation   Session 2:   - Session plan 2: Goal setting - Webpage: Session 2, including activity sheets below, example goals, and video ‘SLT talking about example goals’ - Activity sheet: Goal setting sheet - Activity sheet: Action plan - Activity sheet: My vision - Home activity: reflecting on use of chosen strategies and impact of use   Session 3:   - Session plan 3: Practise using strategies - Webpage: Session 3, including explanation of home activities - Activity sheet: Conversation topic list - Home activity: Send video of conversation using strategies to therapist - Home activity: reflecting on use of chosen strategies and impact of use - Home activity: log use of strategies   Session 4:   - Session plan 4: Reflection on progress and self-monitoring - Webpage: Session 4, including explanation of session and home activities - Activity sheet: Rating scales - Home activity: reflecting on use of chosen strategies and impact of use - Home activity: log use of strategies - Home activity: reflection on conversations with others   Session 5:   - Session plan 5: Using skills and strategies in other situations - Webpage: Session 5, including video ‘Strategies in conversation’ - Information sheet: General strategies in conversation - Homework tasks: plan for how to implement changes and support generalisation beyond the dyad   Session 6:   - Session plan 6: Reviewing and planning for the future - Webpage: Your final session, including progress made, what happens next, and support available - Activity sheet: Summary of your therapy - Activity sheet: After you have finished Better Conversations   Planned: following refinement of the intervention programme and resources, resources will be made freely available via the Better Conversations website |
| 4. | **What**  Procedures:  The SLT holds an interview to explore the nature of conversation and how it works with the person with Parkinson’s and their conversation partner (a friend or family member). Video clips of the dyad having a conversation are used to identify facilitators (strategies that help) and barriers to successful conversation. These video clips are discussed in the sessions with the SLT to identify personal goals for the person with Parkinson’s and their conversation partner. Goals are recorded using the Goal Attainment Scaling framework (Turner-Stokes, 2009). Each member of the dyad identifies target communication strategies which they practise through conversation-based activities, role play and homework tasks. Finally, the SLT explores with the person with Parkinson’s and their conversation partner how to plan for changes in communication and how to employ strategies in different situations and contexts. Following is a summary of the topics/ aims for each session:  Session 1:   - understanding what conversation means, understanding what makes a conversation work well, exploring how dysarthria can affect conversation - build understanding of how conversation works and awareness of own conversation strengths - increase expectations for what the therapy will involve and what might be achieved over the course of therapy   Session 2:   - review of dyad video to gain more in depth understanding of current conversation barriers and facilitators - explore how success of conversation is impacted by context and strategies used - set specific, measurable, achievable, realistic and time-bound goals using the Goal Attainment Scaling framework (Turner-Stokes, 2009)   Session 3:   - observe and reflect upon use of strategies and the effect of using strategies (e.g. on other member of dyad, on flow of conversation, on emotional consequences) - practise/ rehearse using conversation strategies   Session 4:   - review of dyad video to increase self-reflection and review goals - use self-monitoring and feedback to maximise existing strategies and practise building new skills   Session 5:   - Gain a more in depth understanding of how the communication context impacts on the success of conversations - Identify skills that can be used in different contexts (e.g. group settings, outside the house) - explore how to increase the success of conversations with other people, for example through self-advocacy and strategy use   Session 6:   - reviewing and planning for the future: recapping what has been covered in the sessions with a focus on both participants and conversation as a whole (rather than speech) - focus on goals identified in previous sessions, and plan for generalisation and sustainability |
| 5. | **Who provided**  BCP is delivered by a qualified speech and language therapist. |
| 6. | **How**  BCP is delivered remotely to a person with Parkinson’s and their chosen communication partner (e.g. a friend or family member who they regularly converse with). The person with Parkinson’s and their communication partner require access to a WiFi connection to take part. |
| 7. | **Where**  Sessions are delivered remotely via a video conferencing platform, with the dyad at home and the SLT at their place of work. |
| 8. | **When and how much**  The BCP program consists of 6 weekly sessions lasting approximately 60 minutes each. Additional sessions prior to and after the program are required for outcome measurement. |
| 9. | **Tailoring**  The BCP programme is tailored to each individual dyad. Each dyad records three 15-minute video clips of themselves having a conversation, which are then reviewed by the SLT prior to starting the intervention. Short clips are used during therapy as video-feedback to provide examples of positive communication strategies and possible barriers to successful conversation so that the SLT can support each member of the dyad to identify personalised goals for intervention. Session plans suggest opportunities for individualisation, such as choosing to spend time on identifying new goals versus practising existing strategies. |
| 10. | **Modifications**  Planned: we anticipate future modifications will be made in response to feedback from pilot study participants, the SLT administering therapy and future Patient Public Involvement work. |
| 11. | **How well**  Planned: user acceptability was evaluated after each therapy session via feedback forms, and via adherence data. Participants completed speech, communication and quality of life outcome measures prior to and after the intervention, and made two further conversation recordings after BCP. Outcomes have been evaluated and further work is required to identify the most appropriate measures which are sensitive to potential change after BCP. |

**References:**

Hoffmann, T. C., Glasziou, P. P., Boutron, I., Milne, R., Perera, R., Moher, D., Altman, D. G., Barbour, V., Macdonald, H., Johnston, M., Lamb, S. E., Dixon-Woods, M., McCulloch, P., Wyatt, J. C., Chan, A.-W., & Michie, S. (2014). Better reporting of interventions: template for intervention description and replication (TIDieR) checklist and guide. *BMJ : British Medical Journal*, *348*, g1687. https://doi.org/10.1136/bmj.g1687

Johnson, F. M., Best, W., Beckley, F. C., Maxim, J., & Beeke, S. (2017). Identifying mechanisms of change in a conversation therapy for aphasia using behaviour change theory and qualitative methods. *International Journal of Language & Communication Disorders*, *52*(3), 374–387. https://doi.org/https://doi.org/10.1111/1460-6984.12279

Turner-Stokes, L. (2009). Goal attainment scaling (GAS) in rehabilitation: a practical guide. *Clinical Rehabilitation*, *23*(4), 362–370. https://doi.org/10.1177/0269215508101742

Yorkston, K., Baylor, C., & Britton, D. (2017). Speech versus speaking: the experiences of people with Parkinson’s disease and implications for intervention. *American Journal of Speech-Language Pathology*, *26*(2S), 561–568. https://doi.org/10.1044/2017_AJSLP-16-0087
