# Supplementary material for: Better Conversations With Parkinson's: Feasibility and Acceptability of a Novel Communication Partner Training Telehealth Intervention
Source: Int J Lang Commun Disord. 2026 Aug 4;61(5):e70308. doi: 10.1111/1460-6984.70308 (PMC13437927; doi:10.1111/1460-6984.70308)
Supplement: Supplementary file 2 [file JLCD-61-0-s002.docx]

## Supporting Information 5: Examples of SLT interview responses for each Theoretical Framework of Acceptability construct

The theme of ‘Other’ represents comments that were deemed relevant from the interview but did not fit into constructs from the Theoretical Framework of Acceptability.
