# Supplementary material for: Better Conversations With Parkinson's: Feasibility and Acceptability of a Novel Communication Partner Training Telehealth Intervention
Source: Int J Lang Commun Disord. 2026 Aug 4;61(5):e70308. doi: 10.1111/1460-6984.70308 (PMC13437927; doi:10.1111/1460-6984.70308)
Supplement: Supplementary file 3 [file JLCD-61-0-s003.docx]

## Supporting Information 3: Topic guides

Focus group topic guide

Below are key questions, with optional follow-up questions, to allow flexibility within the focus group.

**Aim to explore:**

- facilitators to recruitment
- barriers to recruitment
- SLTs’ understanding of their role when recruiting
- SLTs’ understanding of why people with Parkinson’s did/ didn’t want to take part

Intro (5 minutes)

- thank you
- how focus group will work: 90 minutes – try to keep concise; LD & PC facilitating; recording
- expectations: confidential; no judgement of responses / no right or wrong answers; everybody’s opinions are equally valid

Lead in question (15 minutes)

Tell me about how you invited people to take part in the study?
*Did anyone do it differently?
What about people who weren’t due to have an appointment for a while?
Tell me how it worked for people on the waiting list.
How long do you think it took you? / how much time did it add to your day do you think for each participant?
When explaining the research, what key things did you try to get across?*

Barriers & facilitators from SLT point of view (25 minutes)

How easy did you find it to recruit people to the Better Conversations with Parkinson’s study?

- *How easy was it to remember to ask people in appointments?
  What helped you to remember?
  What got in the way of remembering?*
- *How much time did you find you had in appointments to invite participants to take part in the research?
  Did this feel like the right amount of time?
  How achievable did it feel to fit this time into appointments?*
- *How much support did people need to understand the information about the study?
  How well equipped did you feel to give further explanations about the study?*
- *Recap process of recruitment (leaflet, link to share contact details with research team OR invited by letter).
  Was there any part of this process which proved difficult in particular for you?
  Was there anything about this process which worked well from your point of view?*
- *How easy was it to capture those people who you weren’t actively seeing?
  (on your worklist or on the general waiting list)*
- *How much of a priority did recruitment feel for you/ for the team?*

Perceptions of people with Parkinson’s (25 minutes)

Recap process of recruitment *(leaflet, link to share contact details with research team OR invited by letter).*
Was there any part of this process which you feel didn’t work well for the people you were inviting?
Was there any part of this process which you felt worked particularly well for the people with Parkinson’s?

- *What was people’s initial reaction when you brought up the research?*
- *How did people respond to the length of the leaflets?*
- *How did people respond to having to go online to enter contact details?*
- *When explaining the study to people, did you feel there was a point at which people decided not to take part?*
- *Were there any points at which you felt you were encouraging people to take part?
  Were there any times when you felt you were suggesting or recommending someone didn’t take part? What influenced this decision.*
- *Recap reasons in spreadsheet – were there any other reasons people gave for not wanting to take part in the study?*
- *What reasons did people give for wanting to take part in the study?*
- *Did you perceive that there were any other reasons people didn’t mention explicitly?*
- *How do you think people’s responses would have differed if the therapy was different (e.g. loud therapy/ dysphagia therapy/ different dosage)?*

Closing questions (10 minutes)

Summarise conversation so far.
In terms of trying to recruit people to a conversation-based speech and language therapy study, is there anything you think we’ve missed or not talked about today?
What do people think the main barrier to recruitment was, either from your point of view or from the point of view of the person with Parkinson’s?
What was most helpful in terms of recruiting people to the study (from your point of view or for people with Parkinson’s)?

Topic guide for participant interviews

Below are key questions with follow up questions, used flexibly to explore issues raised by the dyad.

***Introduction***

- *I am a student on the Better Conversations with Parkinson’s project.*
- *Today we will be talking about your experiences of the six sessions of conversation therapy you had with [the SLT]. [The SLT] will not be attending this interview today, so please be open and honest when sharing your opinions. Your feedback will be essential to improving the program and ensuring the therapy is as helpful as possible to future candidates.*
- *Before we get started, do I have your consent to record this meeting? The recording will be stored safely and not shared with anyone outside the research team.*
- *Now, I am going to start asking you both some questions. If at any point you feel like you need a break, please feel free to ask.*
- *Do you have any questions before we start?*

***Section 1: Experiences***

- *Tell me about your sessions with [the SLT]. What do you remember about them?*
  - *You received six speech and language therapy sessions, delivered weekly. These were online sessions. Within these sessions, you worked on your conversations and made videos to reflect on strategies that made conversation easier or more difficult.*
- *Before starting therapy, what were you hoping to get out of the sessions?*
  - *Was the therapy what you expected?*
  - *Did [the SLT] effectively explain the aim of the therapy?*
  - *Were there any surprises that arose from doing the program together?*
- *Before you started therapy, were you aware of the impact Parkinson’s can have on speech, language and communication? Did this change after receiving therapy?*
- *What aspect or aspects of therapy did you find most useful?*
  - *The therapy involved: making and watching back videos of your conversations, homework to complete between sessions, thinking about conversations – what helps and makes them harder, using the BCP website and reading the handouts. Out of these, what did you find most useful?*
- *Is there anything you feel was missing from the program?*
  - *How could it be improved? How/why do you think that would help?*
  - *Is there anything you would have changed about how the therapy was run?*
- *Was there anything you feel was not helpful or needed within the therapy sessions?*

***Section 2: Therapy Materials and Components***

- *How did you find the use of online therapy?*
  - *Have you had any face-to-face speech and language therapy input?*
  - *How did being online compare?*
  - *Has this experience impacted how you would react to being offered interventions via telehealth in the future?*
  - *Did it make a difference not having the SLT in the room with you?*
- *Tell me about how you found the therapy materials used.*
  - *Videos, website, handouts, emails, printed materials, video information on the BCP website*
  - *How useful are these for providing ongoing support?*
- *How well prepared did you feel for this therapy?*
- *What skills or qualities do you think a therapist should bring to this sort of therapy?*
  - *If you were advising a therapist on how to successfully deliver this therapy, what would you say?*
  - *What was it about your therapist that made this therapy successful?*
  - *Did the therapist seem knowledgeable? Flexible? Experienced?*
- *How did you feel after each therapy session?*
  - *Tired? Empowered?*
  - *What made you feel that way?*
  - *Was that a drawback for you?*
  - *What could be done to improve this?*

***Section 3: Effects of Therapy***

- *Tell me about your conversations after therapy. How frequent are they?*
  - *Any difficulties or improvements?*
  - *Who are you conversing with?*
  - *How do the conversations make you feel?*
  - *Can you give examples of difficulties you were having before and what changes the therapy has brought about?*
  - *How has your view of yourself as a communicator changed?*
- *What else, if anything, changed as a result of the therapy?*
  - *How did taking part in the therapy impact your quality of life/social life?*
- *Did therapy impact your relationship?*
  - *How? Can you give an example?*
  - *Did anything change in your wider relationships following therapy?*
  - *Are you more empowered? Sympathetic?*
- *Do you feel the therapy helped you to achieve your goals?*
  - *Was the goal setting process explained well?*
  - *Did you feel involved in the goal setting process?*
  - *How well prepared were you to participate in the program and to set goals with the therapist? Is there any way in which that could have been done differently?*
- *What helped you to achieve your goals? / What got in the way of achieving these goals?*
- *[PlwP], do you think [CP] achieved their goals? Vice versa, [CP] do you think [PlwP] achieved their goals?*

***Section 4: Future Planning***

- *Are there any specific strategies you learnt in therapy that you still use?*
  - *What helps versus hinders in conversation?*
  - *Were any strategies particularly helpful? Why?*
  - *What has got in the way of using these strategies?*
  - *How often are you using this strategy?*
- *Can these strategies be used with other people?*
  - *Have you ever used these strategies with other people (e.g. wider family, friends, strangers)?*
  - *Can you give an example?*
  - *Tell me about your conversations with other people.*
  - *Do you feel able to support other people to change how they behave in conversation?*
- *How well do you feel this therapy has prepared you for conversations now, almost 2 months after therapy finished?*
- *How well do you feel this therapy has prepared you for conversations in the future?*
  - *What could be done to make you feel more prepared?*
  - *Would you be interested in a refresher session?*

***Section 5: Final thoughts***

- *Would you recommend this therapy program to others?*
- *Have you got anything else you would like to say or any final thoughts that you would like to discuss?*

SLT interview topic guide: interview 1

| **Questions** | **Probe** |
| --- | --- |
| *Self-introduction* | |
| *Maybe we should start by introducing ourselves* | My name is … & my name is … We both study to become speech, language, hearing and reading therapists  Interest areas:  Aphasia, Acquired Brain Injury, Dysarthria |
| *Can you tell us a little bit about yourself?* | Do you have any other education than SLT?  Do you have any previous experience with working people with Parkinson’s?  How many years of experience do you have?  Do you have any hobbies that you enjoy pursuing in your free time? |
| *How did you get involved with the BC-P?* |  |
| *What were your initial thoughts of the programme?* |  |
| *What was/is your initial/current role on the BC-P team?* |  |
| *Context (BCP programme)* | |
| *If a speech therapist came through your door and he/she had never heard of BCP, what are the two most important things you would like to tell/highlight about BCP?* | Why did you highlight that specifically? |
| *Can you tell us about the structure of the BCP intervention?* | In what way did the BC programme get adjusted to people with Parkinson’s and their communication partner?  Can you read about the adjustments somewhere? |
| *Who are the candidates for the BC-programme?* | Can people with both types of Parkinson’s (atypical + typical) and perhaps Parkinson’s disease dementia participate in the programme?  Or does it require a certain level of insight, motivation, cognition and/or abilities to benefit from the programme? |
| *What were your initial expectations of the BC-P programme concerning the effectiveness?* | Did your expectations match with the outcome? |
| *What preparations did you need to make before carrying out the intervention?*  *And how much time did you spend post-processing?* | How much time did you spend with practical preparations? And by practical preparations we mean:   - Camera setup - Journal reading - Time - Candidacy      - Initial conversation - Intervention presentation - Research brush up - Journal writing and reading - Video Analysis / Conversation Analysis |
| *Did the preparations to the intervention line up with your initial expectation of the preparation time?* | Was there more/less work required than expected? |
| *From your experience, what were the specific advantages and disadvantages of the BCP programme?* | - Time commitments, timing of appointments - Videotaping, Video Analysis - Telehealth, cognitive advantages and disadvantages, IT capabilities - The length of the session, length of intervention |
| *What was the most challenging part of delivering the intervention?* | - Was it challenging to let the participants discover their own facilitators and barriers? - Not to interfere? |
| Were there anything in particular you liked/disliked about the BCP programme? | - Did anything go against your values? |
| *From your initial understanding of the BC programme (before delivering the intervention) to now has there been any changes in your understanding after delivering the BCP?* |  |
| *What was your experience with the dyads?* | - Motivation before and after they know what the programme is about, and from where/who does the motivation primarily come from? - Cooperation? Resistance? Disease insight and cognition? |
| *Are there any things you would have done differently, given the experience you have now?* |  |
| *Questions about English practice:* | |
| *How much time is usually allocated for training in English practice?* |  |
| *How long are the typical courses / treatment period that are granted?* |  |
| *Is BCP designed so that you can simultaneously work individually with the participant, e.g. with LSVT?* |  |
| *Could any SLT according to you perform the intervention?* |  |
| *Did you receive supervision along the way? Did you find the instructions in the BC manual book helpful? Did you have to seek additional knowledge outside the BCP programme?* |  |
| *Visions for BCP:* | |
| *How much do you think BCP will come to play a role in English speech and language therapy practice?* |  |
| *What are your visions/wishes for the programme?* |  |
| *Should all speech therapists worldwide end up using it?* |  |
| *Outro* | |
| *Thank you for your participation, your time and information*  *Would you like a copy of the transcript to be sent to you for validity checking?*  *Small talk* |  |
| *Until next time 12^th^ September at 2 pm UK time* | |

SLT interview topic guide: interview 2

**Initial greetings and small talk** *Thank you for the previous interview; it was very interesting, and just as interesting to revisit afterward through the transcription, where we captured a few more details.*

*As you know, we are looking into the acceptability of the delivery of the BCP program, so the follow-up questions we would like to ask you today will focus on this. Some are questions you may feel you may have answered already, but they are intended to ensure that we cover all aspects thoroughly.*

**Affective attitude:**
*In our previous conversation you told us about your initial thoughts on the BCP programme, how you thought it was missing and relevant in the clinical pracsis.
How do you feel about the programme now that you have delivered it?
Has there been any changes in your understanding on how the BCP works after delivering the?*

**Burden:**  *What is your perception of the amount of effort that is required to deliver the intervention?
Was that also the amount of effort that you expected prior to delivering the intervention?*

**Ethicality:**
*Do you feel like your personal value system is a good fit with the core concepts of the BCP programme?
If yes in what way?
Did anything go against your values?*

*Was that also a worry prior to delivering the intervention? / was there anything you thought might go against your beliefs prior to delivering the programme?*

**Perceived effectiveness:**
*What were your expectations of the effectiveness of delivering the programme? (did you expect your delivery to help the dyads to reach their intervention goals/ to better their conversations)*

*Was it your impression that the delivery of the programme had that outcome in the end?*

*Did the struggle to recruit people affect the way you delivered the programme - concerning your way of presenting and keeping the participants engaged throughout the program, did the couple that was less interested have any impact on your delivery of the intervention?*

**Other:**
*Do you believe that all SLTs could perform this intervention? - eg. via gathering information from your website, the book: Better Conversations with communication difficulties: a practical guide for clinicians and the training course that you mentioned the last time we spoke.*

*Is there anything you could imagine being different from your experience with delivering the programme versus what other SLTs  may experience?*

*Do you believe that your experience is transferable to other SLTs?*
