# Supplementary material for: Better Conversations With Parkinson's: Feasibility and Acceptability of a Novel Communication Partner Training Telehealth Intervention
Source: Int J Lang Commun Disord. 2026 Aug 4;61(5):e70308. doi: 10.1111/1460-6984.70308 (PMC13437927; doi:10.1111/1460-6984.70308)
Supplement: Supplementary file 4 [file JLCD-61-0-s004.docx]

## Supporting Information 4: Mapping of inductively identified themes onto the Theoretical Framework of Acceptability (TFA)

The table below presents the mapping of themes identified through reflexive thematic analysis onto the seven constructs of the Theoretical Framework of Acceptability (TFA) (Sekhon et al., 2017). The constructs are affective attitude, burden, ethicality, intervention coherence, opportunity costs, perceived effectiveness and self-efficacy. An explanation is provided for how the identified themes link to the TFA.

| **Theme** | **Associated TFA Construct(s)** | **Explanation** |
| --- | --- | --- |
| Changing expectations and beliefs | Intervention Coherence and Perceived Effectiveness | Participants’ initial misunderstandings evolved into clear understanding and appreciation of the intervention’s aims and benefits, reflecting growing intervention coherence and belief in the program’s effectiveness. |
| Being active agents in therapy | Self-Efficacy, Ethicality and Burden | Participants valued having an active role in therapy, which fostered confidence in implementing strategies and demonstrated alignment of the program with their personal values. However, their involvement and sense of responsibility for the intervention’s success also introduced emotional and cognitive burden. |
| Integrating therapy into everyday living | Opportunity Costs and Burden | Participants reported that sessions were generally convenient and accessible yet highlighted difficulties with fatigue and juggling homework with other responsibilities. |
| Degree of (dis)comfort | Affective Attitude and Ethicality | Emotional responses to the program ranged from enjoyment to discomfort, often related to the methods and techniques used (e.g. video recordings of themselves and other people living with Parkinson’s). Despite their discomfort, participants often perceived value in these experiences, demonstrating accordance between the therapy and their values. |
| Needs beyond the therapy | Perceived Effectiveness and Intervention Coherence | Some participants suggested BCP was insufficient or not fit for their needs. Sometimes their accounts raised questions about whether their expectations aligned with what therapy could deliver. |

**References:**

Sekhon, M., Cartwright, M., & Francis, J. J. (2017). Acceptability of healthcare interventions: an overview of reviews and development of a theoretical framework. *BMC Health Serv Res*, *17*(1), 88. <https://doi.org/10.1186/s12913-017-2031-8>
