# Supplementary material for: Better Conversations With Parkinson's: Feasibility and Acceptability of a Novel Communication Partner Training Telehealth Intervention
Source: Int J Lang Commun Disord. 2026 Aug 4;61(5):e70308. doi: 10.1111/1460-6984.70308 (PMC13437927; doi:10.1111/1460-6984.70308)
Supplement: Supplementary file 5 [file JLCD-61-0-s005.docx]

Supporting Information 1: Inclusion and exclusion criteria for potential participants living with Parkinson’s

| Inclusion criteria | a) Over 18 b) Diagnosis of Parkinson’s c) Experiencing intelligibility or communication problems relating to Parkinson’s d) Ability to communicate and understand well enough to enable participation in the BCP programme e) Ability to see and hear well enough to participate in the programme f) Functionally able to engage in the programme (i.e. able to maintain some concentration and remain in a 60-90 minute session; minimal challenging behaviour) g) English as their language of daily use h) Have a communication partner who is able to and consents to participating in the project i) Able and ready to actively participate in conversation therapy at the current time |
| --- | --- |
| Exclusion criteria | a) History of brain lesions or major head trauma b) Major physical illness or disability which could impact on participation c) Major psychiatric diagnosis d) Presents with prominent behavioural disturbance e) Presents with prominent episodic memory, visual memory or visuo-perceptual impairments f) Currently participating in active SLT intervention which may conflict with BCP g) Currently participating or recently participated in research which may conflict with BCP h) No access to Wi-Fi connection to access BCP remotely |
